# Specific Binding Protein ABCC1 Is Associated With Cry2Ab Toxicity in *Helicoverpa armigera*

**DOI:** 10.3389/fphys.2018.00745

**Published:** 2018-06-19

**Authors:** Lin Chen, Jizhen Wei, Chen Liu, Wanna Zhang, Bingjie Wang, LinLin Niu, Gemei Liang

**Affiliations:** ^1^State Key Laboratory for Biology of Plant Diseases and Insect Pests, Institute of Plant Protection, Chinese Academy of Agricultural Sciences, Beijing, China; ^2^College of Plant Protection, Henan Agricultural University, Zhengzhou, China

**Keywords:** *Helicoverpa armigera*, *HaABCC1*, functional receptors, binding, RNAi

## Abstract

A pyramid strategy combining the crystal (Cry) 1A and 2A toxins in *Bacillus thuringiensis* (Bt) crops are active against many species of insects and nematode larvae. It has been widely used to delay pest adaption to genetically modified plants and broaden the insecticidal spectrum in many countries. Unfortunately, Cry2A can also bind with the specific receptor proteins of Cry1A. ATP-binding cassette (ABC) transporters can interact with Cry1A toxins as receptors in the insect midgut, and ABC transporter mutations result in resistance to Bt proteins. However, there is limited knowledge of the ABC transporters that specifically bind to Cry2Ab. Here, we cloned the *ABCC1* gene in *Helicoverpa armigera*, which expressed at all larval stages and in nine different tissues. Expression levels were particularly high in fifth-instar larvae and Malpighian tubules. The two heterologously expressed *HaABCC1* transmembrane domain peptides could specifically bind to Cry2Ab with high affinity levels. Moreover, transfecting *HaABCC1* into the *Spodoptera frugiperda* nine insect cell significantly increased its mortality when exposed to Cry2Ab *in vitro*, and silencing *HaABCC1* in *H. armigera* by RNA interference significantly reduced the mortality of larvae exposed to Cry2Ab *in vivo*. Altogether current results suggest that *HaABCC1* serves as a functional receptor for Cry2Ab.

## Introduction

The crystal (Cry) proteins produced by *Bacillus thuringiensis* (Bt) are specifically toxic to some insect pests, such as Lepidoptera, Diptera, and Coleoptera, while they are almost harmless to non-target organisms (Sanahuja et al., 2011; Pardo-López et al., 2013; Comas et al., 2014; Nicolia et al., 2014). To reduce the use of chemical insecticides, Bt proteins, such as Cry1Ac and Cry2Ab, have been used worldwide as bio-pesticide sprays or expressed in genetically modified (GM) plants to control certain insect pests (Sanahuja et al., 2011; James, 2016). The hectares of Bt crops worldwide increased from 1.1 million in 1996 to 98.5 million in 2016, Bt corn, cotton, and soybean accounted for >99% of this total amount, with a cumulative total of more than 830 million (James, 2016).

The high selection pressure of Bt could lead to the rapid evolution of insect resistance. Cases of pest resistance to Bt proteins produced by GM crops increased from 3 in 2005 to 16 in 2016 (Pardo-López et al., 2013; Jakka et al., 2016; Tabashnik and Carrière, 2017). Bt cotton (expressing the Cry1Ac protein) has been planted in China since 1997, and recent bioassay data showed that the percentage of resistant *Helicoverpa armigera* collected from fields in north China increased from 0.93% (2010) to 5.5% (2013) (Jin et al., 2015). To delay pest adaption to GM crops, some different resistance management strategies have been used, including trait pyramiding (Xue et al., 2008; Brévault et al., 2013; Jin et al., 2015). The “pyramid” strategy has been widely adopted to replace the first generation Bt crops. For example, the transgenic cotton which can produce Cry1Ac and Cry2Ab is the only type of Bt cotton grown in Australia. And it’s also the predominant type of Bt cotton grown in India and the United States (Tabashnik et al., 2013a; Fabrick et al., 2014).

However, the occurrence of cross-resistance sometimes weakens these advantageous characteristics of Cry1A + Cry2A “pyramid” strategy (Tabashnik et al., 2013b; Wei et al., 2015; Welch et al., 2015). Although Cry1A and Cry2A were predicted to have different structures and different mode of actions in the target lepidopteran pests because of their low amino acid homology (Hernández-Rodríguez et al., 2008; Caccia et al., 2010), but studies have indicated that they share some of the same receptors. Several important functional Cry1A receptors, such as cadherin (CAD), amionpeptidase-N (APN), or alkaline phosphatase (ALP), they have been identified as binding protein or reported to play vital functional role in the toxicity of Cry2A (Onofre et al., 2017; Yuan et al., 2017; Zhao et al., 2017). Further research showed although CAD was a functional receptor for both the Cry2Aa and Cry1Ac toxins in *Spodoptera exigua*, but Cry1Ac and Cry2Aa toxins did not compete for the same binding sites and may bind to diverse CAD protein epitopes (Qiu et al., 2015). Thus, the same receptors but different binding sites may associate with the asymmetrical cross-resistance between Cry1A and Cry2A (Tabashnik et al., 2009; Wei et al., 2015). So, it is urgent to find the receptor of Cry2Ab.

Recently, some ATP-binding cassette (ABC) transporters are involved in the resistance of insect to Bt toxins. For example, ABCC2 mutations cause the resistance of *H. armigera* and *Heliothis virescen* to Cry1Ac (Gahan et al., 2010; Xiao et al., 2014). The Cry1Ac-resistance of *Plutella xylostella* is closely related to the reduced expression of *PxABCC2, PxABCC3*, and *PxABCG1* in the midgut (Guo et al., 2015a,b). Meanwhile, high levels of resistance to Bt Cry2Ab toxin have been verified to be genetically linked with loss of function mutations of an ABC transporter gene (ABCA2) in two Lepidopteran insects, *H. armigera* and *H. punctigera* (Tay et al., 2015). Moreover, two *HaABCA2* knockout strains created from the susceptible SCD strain by using the CRISPR/Cas9 genome editing system display high levels of resistance to Cry2Ab (>100-fold) compared with the original SCD strain (Wang et al., 2017). In addition, the binding experiments showed there is more than one receptor of Cry2Ab in insect midgut (Wei et al., 2016). Functional ABC transporter family proteins contain four core domains: two membrane-spanning domains (transmembrane domains, TMs), each built from six membrane-spanning α-helices, alternating with two nucleotide-binding domains (NBDs) located on the cytosolic side (Dassa and Bouige, 2001; Linton, 2007). All of these indicated that other ABC transporters may also involve in the mode of action of Cry2Ab.

ABCC1 has four core domains like other ABC transporter proteins. Additionally, ABCC1 involved in the toxicity of Cry2Ab to *H. armigera* by itraq data (unpublished data). However, whether ABCC1 serves as a functional receptor for Cry2Ab in *H. armigera* remained unclear. Here, we discovered Cry2Ab could bind to the heterologously expressed peptides with high affinity levels, transfecting *HaABCC1* into the Sf9 cell line significantly increased its mortality rate when exposed to Cry2Ab *in vitro*, and silencing *HaABCC1* in *H. armigera* by RNA interference (RNAi) significantly reduced the mortality rates of larvae exposed to Cry2Ab *in vivo*. Based on our results, we propose that ABCC1 in *H. armigera* is involved in the action mode of Cry2Ab.

## Materials and Methods

### Insect Rearing and Tissue Sampling

Susceptible *H. armigera* (96S strain) was collected from the cotton fields in Xinxiang County, Henan Province, China in 1996. The larvae of this colony were reared in the laboratory on an artificial diet without exposure to any Bt toxins or insecticides (Liang et al., 1999).

We collected samples from nine developmental stages of *H. armigera*: egg, first- to sixth-instar larva, pupa, adult (male and female). For each biological replicate, we collected samples from 200 eggs and 5–20 individuals from other developmental stages. The following different tissues: head, foregut, midgut, hindgut, Malpighian tubule, peritrophic membrane, hemolymph, fat body, and cuticle from fifth-instar larvae were collected, and tissues dissected from 25 larvae served as one biological replication. All collected samples were quickly frozen in liquid nitrogen and stored at -80°C for subsequent RNA extraction. Four biological replicates were prepared for each treatment.

### Prepared Cry2Ab and Biotinylated Cry2Ab

The activated Bt toxin Cry2Ab was purchased from Envirologix Inc. (Portland, ME, United States). Activated Cry2Ab protein was biotinylated using the EZ-Link Sulfo *N*-hydroxysuccinimide Liquid Chromatography (LC) Biotinylation Kit (Pierce, FL, United States) with a 1:20 molar ratio (Cry protein:biotin) following the manufacturer’s instructions. Biotinylated Cry2Ab proteins were separated on a 4–20% SDS–polyacrylamide gel, transferred to polyvinylidene difluoride (PVDF) membranes (Millipore Corp., Billerica, MA, United States) (150 mA, 1 h) and were treated in 5% (w/v) bovine serum albumin (BSA) diluted in phosphate buffered saline (PBS; 135 mM NaCl, 2 mM KCl, 10 mM Na_2_HPO_4_, and 1.7 mM KH_2_PO_4_, pH 7.4) containing 0.5% (v/v) Tween-20 (PBST) at 4°C overnight. The membranes were washed three times with PBST, and streptavidin horseradish peroxidase was used to detect biotinylated toxins. The blots were developed with the Easysee Western Blot Kit (Transgen, Beijing, China) and observed in a multifunction laser imager (TYPhoon 9410, GE Healthcare, United States).

### Cloning, Expression, and Purification of *HaABBC1*

#### Total RNA Extraction and First-Strand cDNA Synthesis

TRIzol reagent (Invitrogen, CA, United States) was used to extract total RNA from the midguts of 25 fifth-instar larvae of *H. armigera* according to the manufacturer’s instructions. Then, contaminating genomic DNA was removed by treating with DNase I (TakaRa, Japan). The 260/280 and 260/230 ratios measured using a NanoDrop 3300 (Thermo Fisher, MA, United States) were used to evaluate the purity of the total RNA, and a 1% agarose gel was used to determine the integrity. Then, the first-strand cDNA was synthesized immediately from 1 μg of total RNA using a SuperScript^TM^ III First-Strand Synthesis Kit (Invitrogen) following the manufacturer’s instructions and stored at -20°C for further use.

#### Cloning and Sequence Analysis

Parts of the *ABCC1* gene were obtained from the previous transcriptome sequencing data in our laboratory. Rapid amplification of cDNA ends (RACE) was used to obtain the full-length cDNA. RACE-ready cDNAs were amplified using a SMARTScribe^TM^ RACE cDNA Amplification Kit (Clontech, Mountain View, CA, United States) according to the manufacturer’s instructions. Gene-specific primers were designed based on the fragments of the *ABCC1* gene. The primers were designed by Primer Premier 5.0, and UPM was used as the universal primer (Supplementary Table S1). RACE PCR was used under the following conditions: Initial denaturation at 94°C for 4 min, followed by five cycles at 94°C for 30 s and 72°C for 2.5 min, followed by another five cycles at 94°C, for 30 s, 70°C for 30 s, and 72°C for 2.5 min. Subsequently, 25 cycles were performed at 94°C for 30 s, 68°C for 30 s, and 72°C for 2.5 min, followed by 72°C for 10 min. To ensure the entire open reading frame (ORF) was amplified, specific primers that included initiation and stop codons were designed and used to amplify the entire ORF sequence. PCR products were separated by 1% agarose gel electrophoresis, and the expected bands were gel-purified and cloned into the pEasy-T3 vector (TransGen). The cloned fragments were then sequenced. The full sequence was obtained and submitted to GenBank (accession no. KY796050).

The NCBI BLAST database was used to analyze the homology of the *ABCC1* gene with other ABC sequences (Supplementary Table S2). The molecular weights and isometric points of the proteins were predicted using the ExPaSy proteomics server website^1^. The TM helices were analyzed using TMHMM Server v.2.0^2^. The protein domains were predicted using the ExPaSy-PROSITE^3^. The *N*-terminal signal peptide positions were determined using SignalP 4.1 Server^4^. NetNGlyc was analyzed using NetNGlyc 1.0 Server^5^. The NetOGlyc were analyzed using NetOGlyc 4.0 Server^6^. The percentage of amino acid sequence identity was calculated using ClustalW, and the phylogenetic tree was constructed in MEGA 7.0, using the neighbor-joining method (Tamura et al., 2013).

#### Cloning, Protein Expression, and Purification of Two *HaABCC1* Protein Fragments

Based on the nucleotide sequence of the gene encoding *HaABCC1*, we designed two pairs of primers with EcoRV restriction sites contained in forward primers and HindIII restriction sites contained in reverse primers, to clone two fragments of *HaABCC1* that incorporated the potential toxin-binding regions, which we termed TMD1 and TMD2, respectively. The primers used for the PCR-amplification of TMD1 and TMD2 fragment were TM1-EcoRV-F and TM1-HindIII-R, and TM2-EcoRV-F and TM2-HindIII–R, respectively (Supplementary Table S4).

Then, the synthesized cDNA was used as the template to amplify the two partial fragments by PCR. The PCR program included denaturation at 95°C for 3 min, 35 cycles of denaturation at 95°C for 30 s, annealing at 53°C for 30 s, and extension at 72°C for 90 s, and final extension at 72°C for 10 min. After purified by AxyPrep DNA Gel Extraction Kit (Axygen Scientific, CA, United States), the PCR products were cloned into the pEASY-T3 vector (TransGen Biotech), and then transformed into competent cell, *Escherichia coli* Trans1-T1 cells (TransGen Biotech). The recombinant plasmids were double-digested with *EcoR*V and *Hind*III (TaKaRa, Dalian, China) for 3 h at 37°C, and the products were subcloned into the expression vector pET32a (Novagen, United States), which was digested with the same restriction enzymes to generate the two pET*HaABCC1* recombinant plasmids, which were transfected into *E. coli* BL21 (DE3) cells (Tiangen, Beijing, China) for protein expression.

The two *HaABCC1* proteins fragments were tested by gradient SDS–polyacrylamide gel electrophoresis (PAGE) 4–20% (Genscript Biology Co., China). Then, the two *HaABCC1* proteins fragments were identified by LC-MS/MS (Q-TOF) at the Beijing Protein Institute (Beijing, China).

The expressed *HaABCC1* protein fragments were purified using a micro-protein PAGE recovery kit (Sangon Biotech., Shanghai, China). The two *HaABCC1* fragments were tested for purity using a 4–20% gradient SDS–PAGE (Genscript Biology Co., NJ, United States).

### Ligand Blot Analyses

For the ligand blot analysis, 10 μg purified *HaABCC1* fragments were separated using 4–20% gradient SDS-PAGE gels (Genscript Biology Co., NJ, United States) and then electro-transferred onto PVDF filters (Millipore Corp.). After blocking, the membrane was incubated with biotinylated Cry2Ab toxin (5 μg/ml) in blocking buffer for 1 h at room temperature and then washed three times (15 min each) with PBST. Then, the PVDF membrane was incubated with horseradish peroxidase-conjugated anti-rabbit IgG (ZSGB-BIO, Beijing, China) as a secondary antibody in blocking buffer (1:10,000) for 1 h at room temperature. After additional washing, the membrane was developed using an EasySee Western Blot Kit (TransGen) and exposed on an ImageQuant LAS4000mini system (GE Healthcare, Japan).

### Quantitative Real-Time PCR (qRT-PCR) Analysis

Total RNA extraction and cDNA synthesis of samples from different tissues and developmental stages were performed as described above. The relative mRNA expression levels of the *ABCC1* gene were analyzed by qRT-PCR. The primers can be found in Supplementary Table S3. Each TaqMan qRT-PCR (Tiangen) reaction was performed in a total volume of 20 μl containing the following components: 1 μl template cDNA, 10 μl 2 × SuperReal PreMix (probe), 0.6 μl each 10 μM primer, 0.4 μl 10 μM probe, 0.2 μl 50 × ROX Reference Dye and 7.2 μl RNase-Free ddH_2_O. All qRT-PCR reactions were performed in 96-well optical plates in an ABI 7500 Real-time PCR System (Applied Biosystems). qRT-PCR was performed for 40 cycles of 95°C for 3 s and 60°C for 32 s. Both β-actin (GenBank EU527017) and GAPDH (GenBank JF417983) genes were used internal references, and the expression level of the target gene (ABCC1) for each treatment was normalized with the geometric mean of the expression of the two reference genes (β-actin and GAPDH) (Liu et al., 2015; Zhang et al., 2016). To check reproducibility, six biological replicates were analyzed with each biological replicate consisting of three technical repeats.

### Transient Transfection and Cell Bioassay

The *Spodoptera frugiperda* 9 (Sf9) cell line was cultured in Sf-900 II SFM medium (GIBCO/BRL/Life Technologies) supplemented with 10% heat-inactivated fetal bovine serum (Hyclone-QB perbio, Logan, UT, United States), 50 U/ml penicillin, 50 mg/ml streptomycin, and 12 mg/ml gentamycin (Invitrogen) in an incubator at 28°C (Cordova et al., 2006).

The whole ORF of ABCC1 was cloned into pAc5.1b vector (Huayueyang Biotechnology Co., Ltd, Beijing, China). First, we designed gene-specific primers that contained two restriction enzyme sites. The primers for the PCR-amplification of the ABCC1 ORF were ORF-PmeI-F and ORF-StuI-R (Supplementary Table S4). The PCR reaction and conditions were described in Section 2.4.2. PCR products were cloned into pEasy-T3 vector (TransGen), followed by restriction enzyme digestion with PmeI and StuI (TaKaRa). The pAc5.1b vector was also digested with PmeI and StuI for 3 h at 37°C. The double-enzyme digestion products were electrophoresed on a 1% low-melting point agarose gel (Invitrogen) and the PCR products were purified by AxyPrep DNA gel extraction kit. Then, the *ABCC1* gene was ligated into the pAc5.1b vector for 3 min at 22°C using T4 DNA ligase (Thermo Fisher). The ligation product was transfected into *E. coli* Trans1-T1 cells (TransGen), and individual clones were selected from an LB plate.

First, the activated Cry2Ab was precipitated by adding 4 M acetic acid dropwise to the activation reaction tube to adjust the pH to 8.0. Then, the tube was incubated at 4°C for 30 min and centrifuged at 12,000 × *g* for 20 min. The activated Cry2Ab pellets were washed three times using 40 ml ice cold ddH_2_O, dissolved in 15 ml Sf-900 II SFM media (GIBCO/BRL/Life Technologies) and centrifuged at 12,000 × *g* for 5 min. The upper supernatant was transferred into a clean tube and used as the stock solution for cytotoxicity assays. Finally, the concentrations of activated Cry2Ab toxin in the stock solution were estimated by electrophoresis of 15 μl 200 μg/ml BSA solution as well as 15 μl of Cry2Ab stock solution using SDS–PAGE, and the intensity of the corresponding bands was quantified with Image J software (NIH, v1.46).

Sf9 cells were seeded onto a 12-well plate (∼9 × 10^5^ cells/well), allowing cells to attach overnight (12 h). Then, cells were transiently (5.5 h) transfected with (∼2 mg/well) pAc-ABCC1 plasmid using Cellfectin (Invitrogen; 8 μl per well). The empty pAc5.1b vector was used as the control. The transfection mixture was removed and replaced with 1.5 ml of supplemented medium (Sf-900 II SFM media containing antibiotics and serum). The cells were incubated at 28°C for 60 h. Then, the cells were collected and the cell concentration was measured with a hemocytometer using Trypan blue. In a 96-well micro-plate, 100 μL of cells (∼10,000 cells) were reseeded allowing cells to attach for at least 2.5 h. Using activated Cry2Ab toxin (0.0091 mg/ml) to treat the cells, the mortality was calculated after 5 h (Wei et al., 2016).

### Silencing of *HaABCC1* by RNAi

RNA interference with small interfering RNA (siRNA) was conducted by microinjection to study the role of *HaABCC1* in the susceptibility of *H. armigera* larvae to the Bt toxin Cry2Ab. The *HaABCC1* siRNA and enhanced green fluorescent protein (*EGFP*) siRNA (negative control) used were sequence specific and custom synthesized (Invitrogen). The sequences 5’-GGAUGUACCUGGUGGGCAUTT-3’ and 5’-GCGUUGGGAAGUCAAGUUUTT-3’designed based on the gene-specific TMD region to avoid potential off-target effects were used as siHaABCC1 and siEGFP, respectively.

Two microgram siRNA (siHaABCC1 and siEGFP) by 5 μl-microsyringe (Hamilton, Bonaduz, Switzerland) or 2 μg DEPC water (blank control) was microinjected in the abdomen of the newly emerged third-instar larvae. The injection point was sealed immediately with geoline. In addition, a third parallel non-treated control was performed. Each treatment included 24 individuals and replicated three times. To calculate the RNAi efficiency by qRT-PCR as the above described, 10 larvae were randomly selected for testing at 48 h after the injection.

To evaluate the susceptibility of *H. armigera* to Cry2Ab after RNAi, diet overlay bioassays were used (Hernández-Rodríguez et al., 2008). The toxin was dissolved and diluted in PBS. At the beginning, we distributed 1 ml liquid artificial diet into each well of 24-well plates (TianJin Xiangyushun Co., TianJin, China). After the diet was solidified, 75 μl solution of Bt toxin Cry2Ab (105 μg/cm^2^) and PBS (control) were overlaid on the surface of the artificial diet on two 24-well plates, respectively. Finally, within 48 h of microinjection, 24 third-instar larvae were placed on the surface of the dried diet in the 24-well plates. Each treatment included 24 larvae, and there were three replicates. After 5 days, the numbers of dead larvae were recorded (Zhou et al., 2010).

### Data Analysis

Cell mortality was calculated as the description in Wei et al. (2016). Significant differences among the different treatments were analyzed by one-way analysis of variance (ANOVA), followed by Tukey’s honestly significance difference (HSD) test for mean comparison. All statistical analysis was performed with SPSS v.18.0 (SPSS Inc., Chicago, IL, United States) at *P* < 0.05 level of significance.

## Results

### Cloning, Sequencing, and Phylogenetic Analysis of *HaABCC1*

**FIGURE 1:**
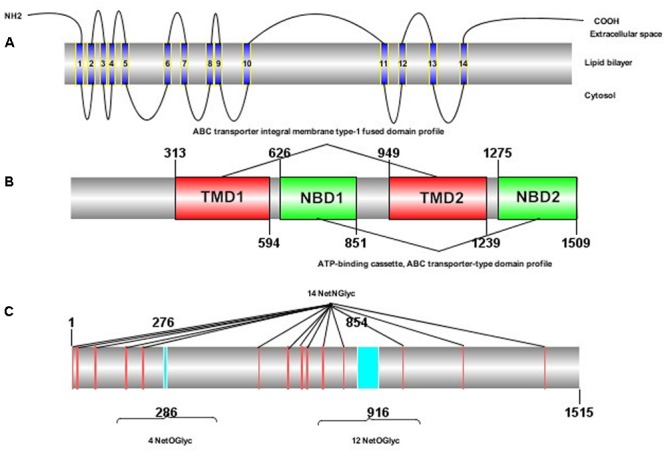
Protein structure of HaABCC1 in *H. armigera*. **(A)** Transmembrane structure of the HaABCC1 protein. The protein contains 14 transmembrane segments. **(B)** Domains of the HaABCC1 protein. The protein contains two ATP-binding cassette (ABC) transporter integral membrane type-1 fused domain profiles (TMD) and two ABC transporter-type domain profiles (NBD). **(C)** NetNGlyc and NetOGlyc sites of the HaABCC1 protein. The protein contains 14 NetNGlyc sites and 16 NetOGlyc sites.

**FIGURE 2:**
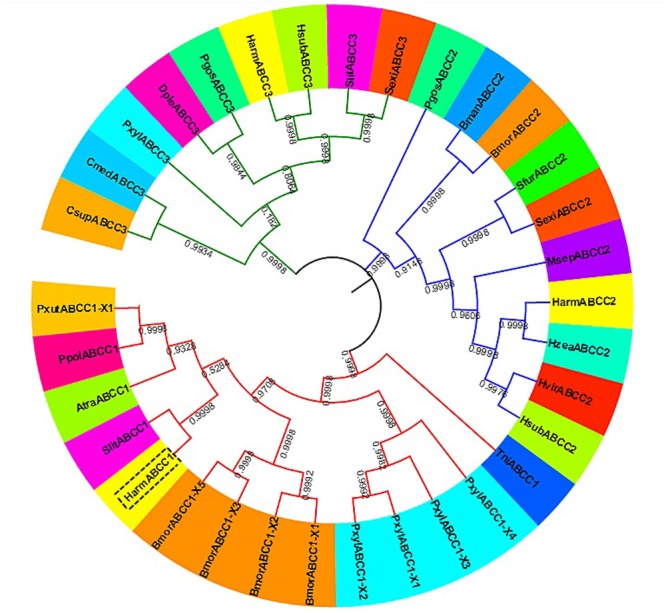
An unrooted phylogenetic tree showing the phylogenetic relationships of ABCC1–3 genes from different insect species. The tree was generated by a ClustalW alignment of the full-length amino acid sequences of ABCC1–3 using the neighbor-joining method in MEGA 7.0. Bootstrap values are expressed as percentages of 1,000 replications. GenBank accession numbers or Gene IDs are displayed in parentheses in Supplementary Table S2.

The ORF of *HaABCC1* (GenBank KY796050) was 4,545 bp, and it encoded predicted proteins of 1,515 amino acid, with a predicted molecular masses of 169.75 kDa. It predicted isoelectric point was 6.68. The protein sequence analysis revealed that *HaABCC1* had no signal peptide. Additionally, it had 14 TM helices, 14 NetNGlyc sites, 16 NetOGlyc sites, and four domains for *HaABCC1* (**Figure 1**). In total, 33 *ABCC1–3* sequences from various species, including those from the current study and those available in GenBank, were aligned and used to construct a phylogenetic tree (**Figure 2**). The phylogenetic tree showed that the ABCC1, ABCC2, and ABCC3 proteins were closely related in different lepidopteran insects. *HaABCC1* was most closely to *SlitABCC1, AtraABCC1, PpolABCC1*, and *PxutABCC1.*

### Spatio-Temporal Expression Pattern of the *HaABCC1* Gene

**FIGURE 3:**
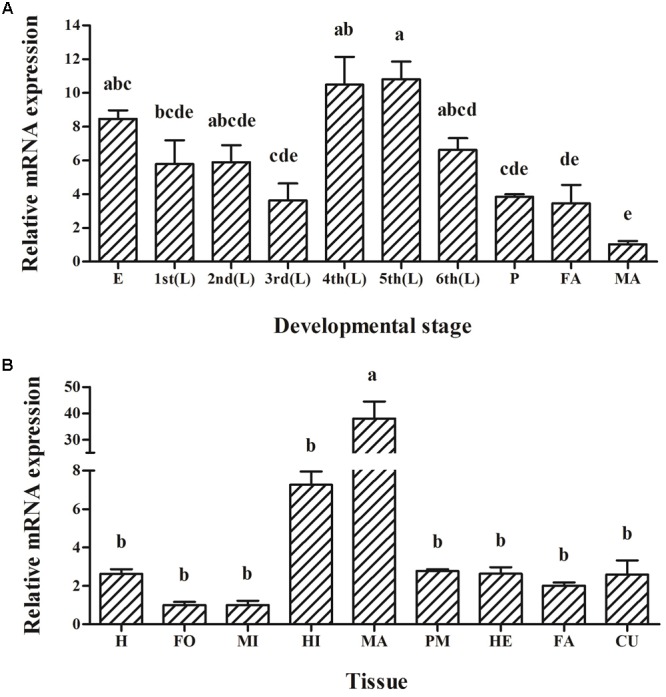
Expression of the *HaABCC1* gene in different developmental stages and tissues of *H. armigera* larvae as determined by a RT-qPCR analysis. **(A)** Relative expression levels of *HaABCC1* in eggs (E), first-instar larvae (1st), second-instar larvae (2nd), third-instar larvae (3rd), fourth-instar larvae (4th), fifth-instar larvae (5th), sixth-instar larvae (6th), pupae (P), female adults (FA), and male adults (MA). **(B)** Relative expression levels of *HaABCC1* in head (H), foregut (FO), midgut (MI), hindgut (HI), Malpighian tubule (MA), peritrophic membrane (PM), hemolymph (HE), fat body (FA), and cuticle (CU) from fifth-instar larvae. Values shown are means and standard errors. Different letters indicate significant expression differences among different tissues or developmental stages based on three biological replications and four technical repeats (*p* < 0.05).

*HaABCC1* was expressed in all developmental stages but peaked in the fourth- and fifth-instar larvae (*p* < 0.001) (**Figure 3A**). Meanwhile, Expression levels of *HaABCC1* were markedly different among the tissues (*p* < 0.001) (**Figure 3B**). The results showed it expressed in midgut, but the highest expression was detected in the Malpighian tubules, for other tissues, the expression levels of *HaABCC1* were relatively low and not significantly different.

### *HaABCC1* Expression, Purification and Ligand Blotting

**FIGURE 4:**
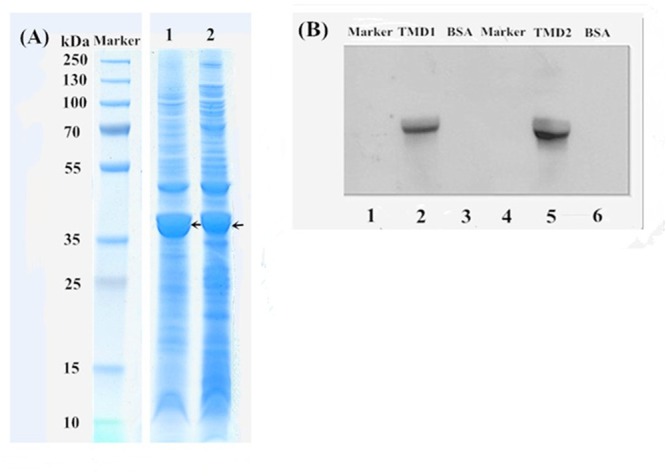
SDS–PAGE and a ligand blot analysis of the HaABCC1 fragments. **(A)** Marker, protein marker; Lane 1, TMD1 fragment after ultrasound treatment; Lane 2, TMD2 fragment after ultrasound treatment. **(B)** Lanes 1 and 4, protein marker; Lanes 3 and 6, bovine serum albumin (BSA), as a control; Lane 2, TMD1 fragment bound with Cry2Ab; Lane 5 TMD2 fragment bound with Cry2Ab.

Two cDNA fragments, TMD1 and TMD2, of the *H. armigera* gene *HaABCC1* (924-bp and 882-bp) were cloned and expressed in *E. coli* BL21 (DE3) cells. In prediction, these cDNA fragments were translated to peptides of 308 and 294 amino acid residues, respectively, of which the molecular weights were 34 and 33 kDa, respectively. All proteins extracted from *E. coli* BL21 (DE3) cells were confirmed by SDS–PAGE (**Figure 4A**), and the recombinant proteins (*HaABCC1*-pET32a construct) were present in the pelleted inclusion bodies. The molecular masses of these proteins were consistent with the predicted molecular weights. The expressed *HaABCC1* protein fragments were purified, and the LC-MS/MS (Q-TOF) indicated that they were parts of the *HaABCC1* protein. To test for specific interactions between the Cry2Ab toxins and *HaABCC1*, a ligand blot analysis was conducted, and the two expressed *HaABCC1* fragments were bound to the activated Cry2Ab. TMD2 had a higher binding affinity than TMD1 (**Figure 4B**).

### Transient Transfection and Cell Bioassay

**FIGURE 5:**
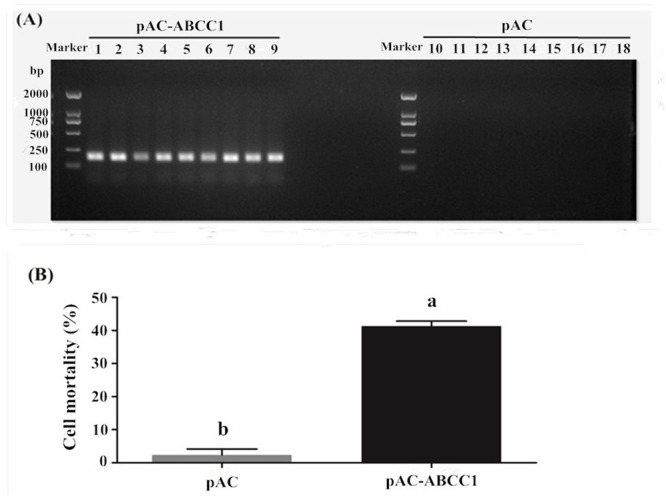
Effects of pAc-ABCC1 plasmid transfection in Sf9 cells on their mortality after being treated with activated Cry2Ab. **(A)** Confirmation of ABCC1 expression in cells transfected with pAc-ABCC1 by PCR. Lines 1–9: pAc-ABCC1 plasmid transfected into the Sf9 cell line (3 biological replicates × 3 technical replicates). Lines 10–18: pAC empty plasmid transfected into the Sf9 cell line. **(B)** The mortality rate of Sf9 cells exposed to Cry2Ab was after being transfected with the pAc-ABCC1 or pAC empty plasmid. Different letters indicate significant differences between treatments (*p* < 0.05).

*HaABCC1* was expressed after be transfected into Sf9 cells (**Figure 5A**). The additional expression of *HaABCC1* led to the Cry2Ab susceptibility significantly increasing, with 41.25% cells dying after being treated by 0.0091 mg/ml Cry2Ab. The mortality rate was significantly greater than that of the control (2.35%) (**Figure 5B**).

### Knockdown of *HaABCC1 in Vivo*

**FIGURE 6:**
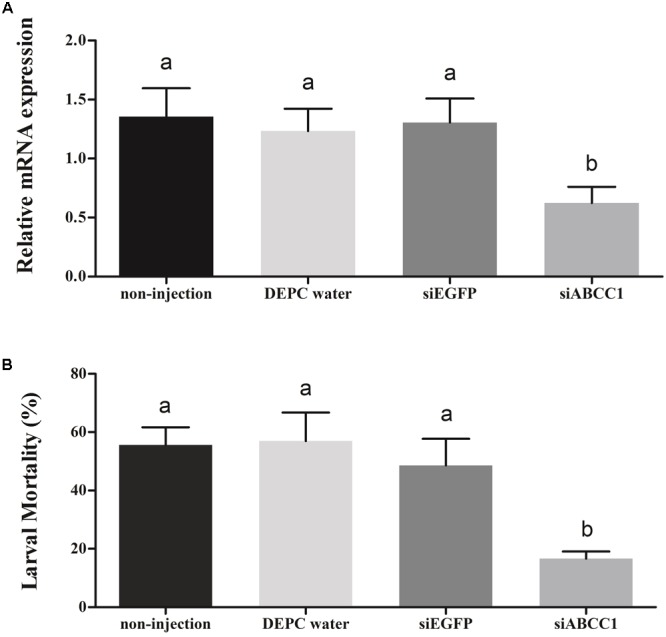
Silencing of *HaABCC1* expression and its effects on *H. armigera* susceptibility to Cry2Ab. **(A)** Effects of the injection of *H. armigera* larvae with DEPC water, siRNA-targeting (si) EGFP, or siABCC1 on the relative expression of *HaABCC1* at 48 h post-RNAi treatment. A non-injected control was also used. Different letters indicate significant differences among treatments (*p* < 0.05). **(B)** Susceptibility of *H. armigera* third-instar larvae to active Cry2Ab toxin as affected by prior injection with DEPC water, siEGFP, or siABCC1. A non-injected control was also used. Different letters indicate significant differences among treatments (*p* < 0.05).

To further explore the potential function of the *HaABCC1* gene in the action mode of Cry2Ab against *H. armigera*, we used *in vivo* RNAi to knockdown *HaABCC1* expression by injecting siABCC1 into early third-instar larvae. The siABCC1 sequence was complementary to the internal gene-specific TMD region of the *HaABCC1* mRNA. The qPCR analysis showed that there were no significant differences among controls (injections of DEPC water and siEGFP, and the non-treated control). Additionally, the injection of siABCC1 into larvae significantly reduced *HaABCC1* transcript levels by 54.0, 49.4, and 52.2% relative to the non-treated, DEPC water- and siEGFP-injected larvae, respectively (*p* < 0.001) (**Figure 6A**). When larvae pretreated with siRNA of *HaABCC1* for 2 days were transferred to a diet containing Cry2Ab, the larval mortality rate of the Cry2Ab-treated group decreased by 70.0, 70.7, and 65.7% relative to non-treated, DEPC water- and siEGFP-injected larvae, respectively. Moreover, there were no significant differences among the three controls (*p* > 0.05) (**Figure 6B**).

## Discussion

Recently, ABC transporters, especially members of the ABCA, ABCB, ABCC, ABCG, and ABCH subfamilies, have become a focus of research in arthropods because of their important roles in xenobiotic transport and insecticide resistance (Dermauw and Van Leeuwen, 2014; Merzendorfer, 2014; Xiao et al., 2014; Guo et al., 2015a,b; Tay et al., 2015). The *HaABCC1* in our study had a structure similar to those of other ABC transporters, containing two extracellular domains that were present as long loops between helices TM I and TM II, 14 NetNGlyc sites and 16 NetOGlyc sites (**Figure 1**). Moreover, its sequence was similar to *SlitABCC1, AtraABCC1, PpolABCC1*, and *PxutABCC1* (**Figure 2**). *HaABCC1* expression was widespread in the *H. armigera* larvae (**Figures 3, 4**), with the highest expression level detected in fourth- and fifth-instar larvae, and in Malpighian tubules, which are part of the excretory and osmoregulatory systems in insect larvae. It was confirmed that ABCC1 was expressed highest in Malpighian tubules in *H. armigera* larvae (Bretschneider et al., 2016). Thus, *HaABCC1* may have functions similar to those of ABC transporters involved in Bt intoxication.

For Cry1A toxins, a sequential mode of action had been proposed, and some specific and saturable binding membrane targets on the midgut are important for the toxicity (Bravo et al., 2011; Heckel, 2012). The toxin first binds to membrane-bound glycosylated proteins, such as aminopeptidases, ALP, and other glycoproteins, and then binds to the 12-CAD domain protein, resulting in processing and accelerated oligomerization (Bravo et al., 2011; Vachon et al., 2012). Cry2A proteins have three domain structures comparable to those of Cry1A toxins (Pigott and Ellar, 2007; Hernández-Rodríguez et al., 2008), making them likely to act in similar ways as pore-forming toxins. Upon activation, Cry2A toxins can bind to the glycosylated loops of TMD1 and/or TMD2 in ABC transporters of *Helicoverpa* species, and this binding can form the basis of oligomerization and bring the pre-pore structures close to the TMDs for pore insertion (Hernández-Rodríguez et al., 2008; Gahan et al., 2010). Additionally, other proteins may be involved in Cry2Ab binding and pore formation, particularly because mammalian ABCs may occur in multi-protein complexes in the membrane (Kaminski et al., 2006). In our study, we also found the two expressed *HaABCC1* fragments (TMD1 and TMD2) could bind with the activated Cry2Ab as assessed by ligand blotting experiments (**Figure 4B**). We hypothesized that ABCC1 may also provide binding and pore insertion functions in the action mode of Cry2Ab, like other ABC transporters.

In fact, some ABC transporters, like ABCC2, ABCC3, and ABCG1 etc. have been proved as functional receptors for Cry1A (Xiao et al., 2014; Guo et al., 2015a,b; Tanaka et al., 2016). For example, the over-expression of *SlABCC3* strongly increases the susceptibility of *Trichoplusia ni* Hi5 cells to Cry1A, which suggested that ABCC3 was also a functional receptor of Cry1A toxins (Chen et al., 2015). Additionally, the ABC transporter mutations have been identified as being associated with Bt resistance. ABCC2 in *H. virescens* were first reported as being involved in Cry1Ac resistance based on quantitative trait locus (QTL) mapping results that investigated the insertion of a premature stop codon in ABCC2 (Gahan et al., 2010). Then, different deletions, point mutations, truncations and spliceosome variants in ABC transporter orthologs were subsequently reported as being associated with the resistance of *P. xylostella, T. ni*, and *H. armigera* to Cry1Ac or Cry2Ab (Baxter et al., 2011; Xiao et al., 2014; Tay et al., 2015), *Bombyx mori* to Cry1Ab (Atsumi et al., 2012), and *Ostrinia nubilalis* survival on transgenic Cry1Fa maize (Coates and Siegfried, 2015). Furthermore, the changes in ABCC2, ABCC3, or ABCG1 transcript levels or their down-regulation were also linked to Cry1A or Cry1Ca toxin-resistance in *S. exigua, P. xylostella*, and *Ostrinia furnacalis* (Park et al., 2014; Guo et al., 2015a,b; Zhang et al., 2017). Here, we provided evidences that *HaABCC1* encoded a functional receptor for Cry2Ab in *H. armigera*. We tested the susceptibility changes in *in vitro* experiments after transfecting *HaABCC1* into Sf9 cells, and the transfected cells were more susceptible to Cry2Ab (**Figure 5B**). To confirm this view, we knocked down *HaABCC1* expression using RNAi technology, and the larval mortality significantly decreased (**Figure 6B**). The specific and saturable binding to membranes in *Helicoverpa* species has been shown for Cry2Ab (Hernández-Rodríguez et al., 2008, 2013), and the resistance to Cry2Ab is also associated with a loss of binding between receptors and Cry2Ab (Caccia et al., 2010). However, the function of ABCC1 in the resistance evolution of *H. armigera* to Bt requires further research. Meanwhile, ABCC1 and ABCC2 are belonging to ABC transporter C family members. ABCC2 is a functional receptor of Cry1Ac and the mutations of it caused the resistance to Cry1Ac. But here we first report ABCC1 is a functional receptor of Cry2Ab. So, whether ABCC1 (or ABCC2) have function in mode of action of Cry1Ac (or Cry2Ab), and caused the cross-resistance between Cry1Ac and Cry2Ab, then reduced the benefit of Cry1A + Cry2A “pyramid” strategy, which are also requires further study.

## Conclusion

This is the first report to demonstrate that ABCC1 is associated with Cry2Ab toxicity in *H. armigera*. The results presented here indicated that HaABCC1 could bind with the Cry2Ab toxin and had important roles in the action modes of Cry2Ab. We suggested that ABCC1 serves as a functional receptor in *H. armigera* for Cry2Ab. Our findings provide mechanistic insights into the interactions between ABCC1 and Cry toxins.

## Author Contributions

GL and LC designed the study. LC prepared experimental materials, performed the experiments, and analyzed the data. GL, LC, and JW wrote the manuscript. CL, WZ, BW, and LN contributed to completing the experimental contents. All authors have read and approved the manuscript for publication.

## Conflict of Interest Statement

The authors declare that the research was conducted in the absence of any commercial or financial relationships that could be construed as a potential conflict of interest.

## Footnotes

1 http://www.expasy.org

2 http://www.cbs.dtu.dk/services/TMHMM/

3 http://prosite.expasy.org/

4 http://www.cbs.dtu.dk/services/SignalP/

5 http://www.cbs.dtu.dk/services/NetNGlyc/

6 http://www.cbs.dtu.dk/services/NetOGlyc/
