# Supplementary material for: Specific Binding Protein ABCC1 Is Associated With Cry2Ab Toxicity in Helicoverpa armigera
Source: Front Physiol. 2018 Jun 19;9:745. doi: 10.3389/fphys.2018.00745 (PMC6018205; doi:10.3389/fphys.2018.00745)
Supplement: Supplementary file 1 [file Table_1.PDF]

Supplementary Material

Specific Binding Protein ABCC1 is Associated with Cry2Ab Toxicity in *Helicoverpa armigera*

Lin Chen[1], Jizhen Wei[2], Chen Liu[1], Wanna Zhang[1], Bingjie Wang[1], LinLin Niu[1], Gemei Liang*[1]

*** Correspondence:** Gemei Liang: gmliang@ippcaas.cn

Supplementary Data

Table S1 Nucleotide primers used to obtain the full-length cDNA of *HaABCC1* by degenerate and RACE PCR

Primer	Sequence (5'- 3')
Degenerate PCR	
HaABCC1PD-F	CCCAATTTCAATCGAGAACG
HaABCC1PD-R	TGGCATAAGGTCAGCGAAAA
HaABCC1ORF-F	ATGTCTTACAATTCTACGCTT
HaABCC1ORF-R	TTAATTAACCAGTCCGGCG
RACE PCR	
HaABCC1RACE-F	TTGGTGAACGGAACTGTGGA
HaABCC1RACE-R	GCTAAGATGGTGAAGCAGGAA

Table S2 The names and accession numbers of genes used in the phylogenetic tree analysis

Gene name	Species	Accession number

Gene name	Species	Accession number
HarmABCC1	*Helicoverpa armigera*	ARE31046.1
AtraABCC1	*Amyelois transitella*	XP_013183079.1
BmorABCC1-X1	*Bombyx mori*	XP_012546132.1
BmorABCC1-X2	*Bombyx mori*	XP_012546133.1
BmorABCC1-X3	*Bombyx mori*	XP_012546134
BmorABCC1-X5	*Bombyx mori*	XP_012546136.1
PpolABCC1	*Papilio polytes*	XP_013142439.1
PxutABCC1	*Papilio xuthus*	XP_013177998.1
PxylABCC1-X1	*Plutella xylostella*	XP_011561541.1
PxylABCC1-X2	*Plutella xylostella*	XP_011561542.1
PxylABCC1-X3	*Plutella xylostella*	XP_011561543.1
PxylABCC1-X4	*Plutella xylostella*	XP_011561544.1
SlitABCC1	*Spodoptera litura*	AKC34057.1
TniABCC1	*Trichoplusia ni*	ADB45217.1
BmanABCC2	*Bombyx mandarina*	AFI44049.1
BmorABCC2	*Bombyx mori*	BAK82126.1
HarmABCC2	*Helicoverpa armigera*	AHL68986.1
HzeaABCC2	*Helicoverpa zea*	AKH49600.1
HsubABCC2	*Heliothis subflexa*	ADH16744.1
HvirABCC2	*Heliothis virescens*	ADH16740.1

Gene name	Species	Accession number
MsepaABCC2	*Mythimna separata*	AJD79136.1
PgosABCC2	*Pectinophora gossypiella*	AJD79134.1
SexiABCC2	*Spodoptera exigua*	AIB06821.1
SfruABCC2	*Spodoptera frugiperda*	ASA45739.1
CsuoABCC3	*Chilo suppressalis*	AJD79133.1
CmedABCC3	*Cnaphalocrocis medinalis*	ASU47346.1
DpleABCC3	*Danaus plexippus plexippus*	OWR51776.1
HarmABCC3	*Helicoverpa armigera*	AHL68987.1
HsubABCC3	*Heliothis subflexa*	ADH16743.1
PgosABCC3	*Pectinophora gossypiella*	AJD79132.1
PxylABCC3	*Plutella xylostella*	AKC96181.1
SexiABCC3	*Spodoptera exigua*	AIB06823.1
SlitABCC3	*Spodoptera litura*	AKC34055.1

Table S3 Nucleotide primers used for qRT-PCR

Primer	Sequence (5'- 3')
GAPDH-F	CATTGAAGGTCTGATGACCACTGT
GAPDH-R	CAGAGGGTCCATCCACTGTCTT
GAPDH-Probe	CACGCCACCATTGCCACCCA
β-actin-F	GGCCCCGTCCACAATGA

Primer	Sequence (5'- 3')
β-actin-R	CCGATCCATACGGAGTACTTCCT
β-actin-Probe	ATCAAGATCATCGCGCCCCCAGA
ABCC1-F	GGCAGCGTGAAAAGAAAGAC
ABCC1-R	GTGATCACGGACGAGAGGAT
ABCC1-Probe	CGAAGCTGAGAAAACCGAGACTGGAAG

Table S4 Nucleotide primers used to obtain the TMD1 and TMD2 fragments and the ORF of ABCC1

Primer	Sequence (5'- 3')
TM1-EcoRV-F	GATATCTCGTTCGGGTGCTTTGTACTG
TM1-HindIII-R	AAGCTTAGATTCCGACGCTCATCAAA
TM2-EcoRV -F	GATATCGCGTTCGGCGGTCAGTTC
TM2-HindIII -R	AAGCTTACGTGACATCGAGTTCATCACAATT
ORF-PmeI-F	GTTTAAACATGTCTTACAATTCTACGCTTG
ORF-StuI-R	AGGCCTTTAATTAACCAGTCCGGCGTCC
